# A Cell-Based Model for Quorum Sensing in Heterogeneous Bacterial Colonies

**DOI:** 10.1371/journal.pcbi.1000819

**Published:** 2010-06-17

**Authors:** Pontus Melke, Patrik Sahlin, Andre Levchenko, Henrik Jönsson

**Affiliations:** 1 Computational Biology and Biological Physics, Department of Astronomy and Theoretical Physics, Lund University, Lund, Sweden; 2 Department of Biomedical Engineering, Johns Hopkins University, Baltimore, Maryland, United States of America; Princeton University, United States of America

## Abstract

Although bacteria are unicellular organisms, they have the ability to act in concert by synthesizing and detecting small diffusing autoinducer molecules. The phenomenon, known as quorum sensing, has mainly been proposed to serve as a means for cell-density measurement. Here, we use a cell-based model of growing bacterial microcolonies to investigate a quorum-sensing mechanism at a single cell level. We show that the model indeed predicts a density-dependent behavior, highly dependent on local cell-clustering and the geometry of the space where the colony is evolving. We analyze the molecular network with two positive feedback loops to find the multistability regions and show how the quorum-sensing mechanism depends on different model parameters. Specifically, we show that the switching capability of the network leads to more constraints on parameters in a natural environment where the bacteria themselves produce autoinducer than compared to situations where autoinducer is introduced externally. The cell-based model also allows us to investigate mixed populations, where non-producing cheater cells are shown to have a fitness advantage, but still cannot completely outcompete producer cells. Simulations, therefore, are able to predict the relative fitness of cheater cells from experiments and can also display and account for the paradoxical phenomenon seen in experiments; even though the cheater cells have a fitness advantage in each of the investigated groups, the overall effect is an increase in the fraction of producer cells. The cell-based type of model presented here together with high-resolution experiments will play an integral role in a more explicit and precise comparison of models and experiments, addressing quorum sensing at a cellular resolution.

## Author Summary

Unicellular organisms have the ability to communicate with each other via signaling molecules, leading to correlated behaviors resembling that of higher organisms. This process, called quorum sensing, allows the cells to monitor the population size or density in a decentralized fashion and perform a common task when these parameters exceed predefined threshold values. The quorum sensing mechanism has been implicated in diverse functions such as producing bioluminescence, virulence factors, and initiating biofilm formation. Complex emergent behaviors, such as quorum sensing, can be hard to analyze and understand without the assistance of mathematical and computational models. Here, we present a cell-based model of proliferating bacterial microcolonies and investigate how population-level responses can emerge from the signaling and mechanical properties of individual cells. We study both signaling variations within homogeneous (homotypic) bacterial populations as well as signaling and competition in mixed heterotypic populations. We investigate in particular how population size, local cell density, and spatial confinement affect colony growth and predict strategies for facilitating quorum sensing. We also show that the interplay between “honest” quorum sensing signal producing bacteria and non-producing “cheaters” can lead to emergent feedback regulation via differentiated growth that provides only a transient benefit for cheating cells.

## Introduction

Bacteria have evolved signaling networks enabling them to sense the environment by producing, exporting and importing small signaling molecules called autoinducers. By using autoinducers that can rapidly diffuse across cell populations and accumulate over time, bacterial cells can receive information about the cellular density in the surrounding environment. The information can then be used to generate decentralized population-wide responses at high enough cell densities. This phenomenon, known as quorum sensing (QS), has been shown to be important for several biological mechanisms since the initial discovery of it as a regulator of bioluminescence [1]–[3]. In particular, it appears to be a key regulator of several bacterial phenotypes with medical implications, e.g. virulence factor production, biofilm development, and synthesis of antibiotics [4]–[6].

**Figure 1:**
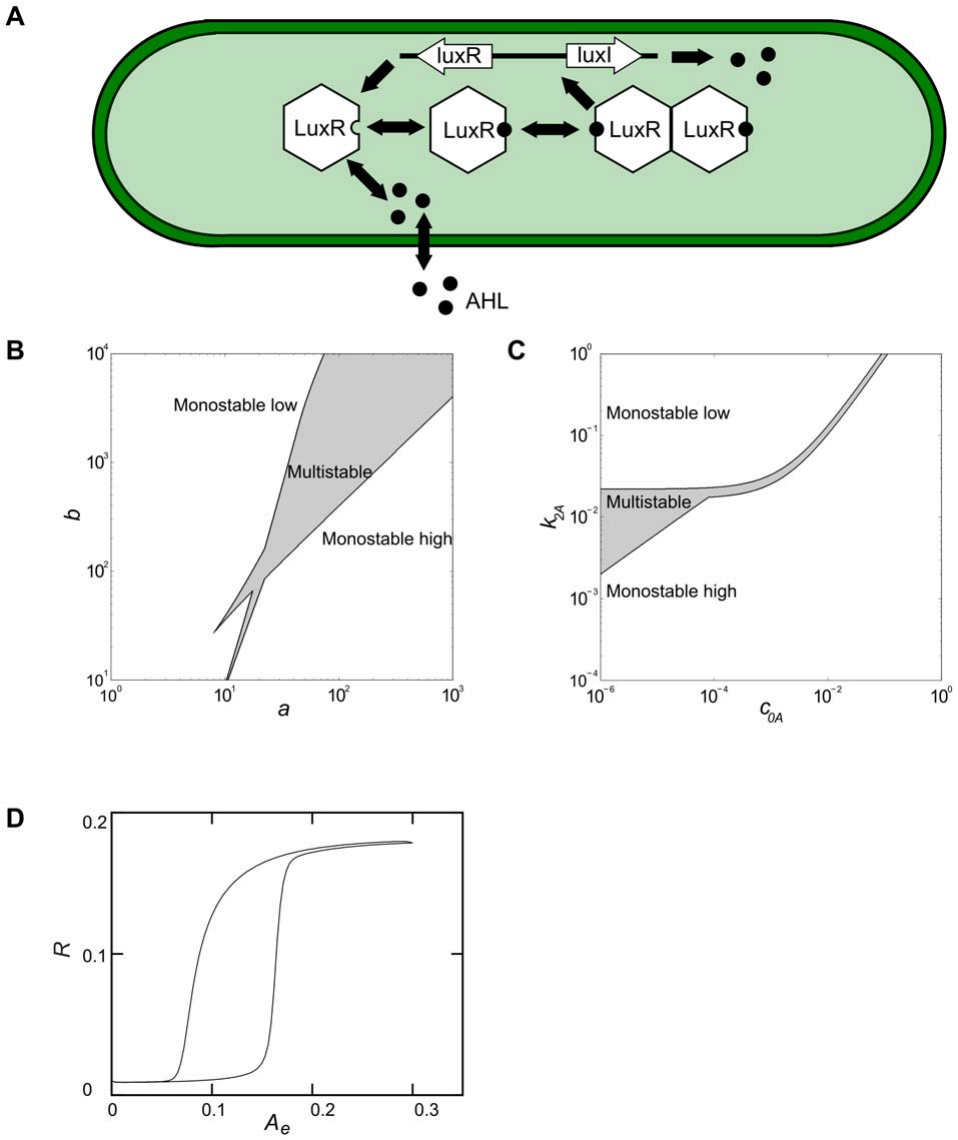
The quorum sensing network. **A**) Illustration of the quorum-sensing network used in our simulations. The autoinducer, AHL, can penetrate the cell wall and bind to and activate LuxR. The activated LuxR forms complexes which in turn affect the synthesis of both the autoinducer and LuxR. **B**) Bifurcation diagram of the equilibrium solutions for the single-cell model without AHL transport (Equation 6). The plot shows a plane where 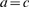 and 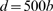, where 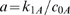, 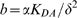, 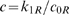, 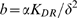, 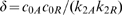, and 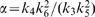. **C**) Bifurcation diagram of the equilibrium solutions for the single-cell model without AHL transport in the modified version where the original parameters were kept (Equation 8 in Methods). The values of 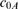 and 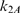 were varied and while the other parameters remained constant. **D**) Colonies display hysteretic behavior in response to changes in external autoinducer level. Shown is the mean concentration of 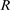 as a function of the external autoinducer concentration 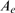. Data are from simulations of a growing colony with bacteria removed once they are outside the simulation boundary, keeping the number of bacteria approximately constant (Suppl. Video S1). The volume of the extracellular medium is assumed to be much larger than that of the bacteria, resulting in negligible effect of cell-produced autoinducer. Standard deviations are smaller than the symbols used in graphs.

Typically, quorum-sensing Gram-negative bacteria use largely homologous quorum-sensing networks [3], wherein the autoinducers are acylated homoserine lactones (AHL), detected and regulated via the genetic circuits similar to the LuxIR circuit in *Vibrio fischeri* (Figure 1A). The lux operon in *V. fischeri* is positively regulated by AHL, and apart from controlling bioluminescence, it upregulates the expression of the AHL-synthase LuxI. This creates a positive feedback loop that increases AHL production in an AHL-sensitive fashion. LuxR is an AHL-dependent luxI activator, whose dimerized complex with AHL leads to transcriptional activation of the operon [7], [8]. LuxR has also been implicated in regulation of its own expression [9]–[11], providing an additional positive feedback loop in the system. The lux operon circuit may be regarded as the central network for controlling QS behavior, but other regulatory mechanisms have also been identified (see e.g. [12], [13]).

Studying QS in detail at a population level introduces some interesting complications. The internal concentration of the autoinducer is dependent not only on its production and degradation, but also on the permeability of the bacterial cell wall as well as on the diffusive properties of the surrounding medium. While the response switch from a low lux gene expression state (off state) to its high expression state (on state) is easily predictable in experiments where the exogenous autoinducer concentration is controlled, the cell response in the presence of autoinducer auto-regulation is more complex to analyze and understand. For instance, waves of QS signaling might develop or be arrested, depending on the mutual location of signaling cells, as the probability for a cell to be induced might depend on the transport properties of the medium and the signaling levels of the neighboring induced cells. The intracellular switch of the QS network is dependent on the autoinducer concentration just outside the cell, and since this concentration increases with the number of nearby cells even if they are in the basal “off” state, the QS can be switched at high densities of bacteria. However, the autoinducer levels are highly dependent not only on the population size, but also on the degree of local cell clustering and on the geometry of the environment in which the bacteria are growing. Since these parameters are not controllable by the individual bacteria, there is an ongoing discussion as to whether the main benefit derived by cells in QS is from measuring cell density (or reaching a “quorum”), the diffusion of autoinducer away from the cell (diffusion sensing, DS) or the potential efficiency of a process metabolically more expensive than secretion of AHL (efficiency sensing, ES) [14], [15].

QS can be beneficial from a population perspective, but since there is a cost associated with ensuring a new beneficial trait for the colony, it is exploitable by the so-called cheater cells, e.g., those that do not contribute to the production of autoinducer or expression of the QS-regulated operon, but still take advantage of whatever benefit the QS response provides to the colony [15]–[20]. This has recently been highlighted in experiments with mixed populations [21], [22]. These experiments have measured the relative fitness of cheater cells, depending on the initial ratio of producer and cheater cells within the colony [21]. In particular, an example of Simpson's paradox was seen [22], wherein QS signal producing cells taken together have a net advantage if cell populations form groups with different initial ratios of producing and cheater cells, even though the producing cells are at a disadvantage in each of the individual groups.

Several mathematical models have been used to describe the molecular network central for quorum sensing [23]–[26]. The models have all used networks with single or double positive feedback loops, and assumed different regulatory mechanisms of luxI via the AHL-LuxR complex. Despite the differences, the models converge in their predictions of a bistable switch-like behavior dependent on the external concentration of the autoinducer. Although the models have provided information on how the intracellular QS-network behaves, the effect at the population level have thus far been excluded in all computational investigations.

To be able to investigate the behavior of quorum sensing in a bacterial colony where the autoinducer is produced within the colony, we introduce a model that explicitly includes growing bacteria interacting with each other and the surrounding environment via both molecular and mechanical interactions. The model assumes two positive feedback mechanisms where a dimerized LuxR-AHL complex activates both LuxI and LuxR production similar to recently published models [25], [26] (Figure 1A). We use a combination of analytical and numerical investigations of the model to explore how for example colony size, local clustering, and confinement, affects the behavior both on the single-cell level as well as on the colony level. In mixed population simulations we investigate the competition between autoinducer producing cells and non-producing cheater cells.

## Results

We developed a mixed cell-based/ODE-model for molecular and mechanical interactions. In the model (see Methods) bacteria are described by two half-spheres connected by a cylinder. The bacteria grow in the direction of the cylinder and divide perpendicular to this direction. Mechanical interactions are explicitly modeled and will tend to minimize spatial overlap in the colony [27]. The molecular network of individual bacteria has two feedback loops. The autoinducer AHL (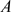) and the receptor LuxR (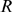) form a dimerized complex that regulates the production of both 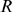 and 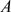 (Figure 1A). The intracellular molecular regulation model is closely based on the model by Williams et al. (2008) [26], where additional dynamics for the autoinducer have been added, as described by the following equations
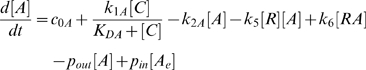
(1)

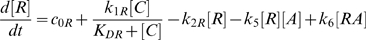
(2)

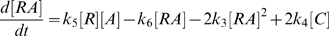
(3)

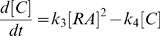
(4)

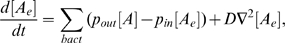
(5)where 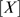 denotes the concentration of a particular molecular species 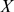, 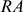 is the AHL-LuxR complex, 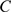 is the dimerized complex, and 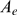 is the extracellular AHL concentration which is assumed to diffuse freely (see Table S1 for parameters). In the cases where growing bacteria were analyzed, the effect of dilution due to the exponential growth was taken into account, giving the equivalence of an extra degradation term in the above equations (see Methods).

### Transport and external autoinducer concentrations can regulate the intracellular switch

First we analyzed the single-cell system described in Equations 1–4 without transport, i.e. 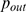 and 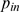 are set to zero. At the steady state, all derivatives are equal to zero, which gives a set of algebraic equations, which in turn can be simplified into a single equation (Equation 6 in Methods), which can have one or multiple positive real roots. Equation 6 was solved numerically to create bifurcation diagrams in the model parameters (Figure 1B and C). It is clear that within a certain parameter region the system has multiple stable solutions, but this region can be complex with several surrounding monostable regions.

Adding intercellular transport and external diffusion is expected to affect the parameters in Equation 6, so that the system trajectory would be able to move into and out of the multistability region(s) (cf. Figure 1B and C). Several single-cell QS models have predicted an 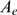-dependent switch-like response of the QS network [23]–[26]. To address the effect of communicating AHL with the cell environment, we first assumed a constant 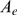 and added transport terms to see how this would affect the equilibrium behavior. This generated two important differences as compared to the non-transport analysis above. The transport out of the cells (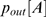 term in Equation 1) has the same form as the degradation (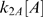), so an increase in outwards transport moves the state of the system towards a monostable “off” state in our equilibrium analysis (upwards in in Figure 1C). The transport into the cells (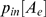) gives an 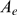 dependent constant contribution and will hence effectively increase the 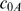 constant which will move the state towards a monostable “on” state (right in Figure 1C). Hence, the addition of transport terms affects the control parameter values and results in changes with opposite effects. At low extracellular AHL concentrations the outflux can dominate the influx and thus drive the bacteria towards an “off” state, whereas high extracellular AHL concentrations are expected to drive the bacteria towards the “on” state.

Note that the analysis above was only for the equilibrium behavior and to investigate the dependence on the external autoinducer concentration in a dynamically growing cell-based model, we next performed simulations wherein 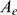 first slowly increased, and then decreased (Video S1). As expected, the colony displayed QS response hysteresis (Figure 1D). For the parameters used here the transition between states was fairly smooth, but for other parameter values the transition can be steeper and can even be irreversible (Figure S1).

### Quorum sensing is a population size effect

The requirement to have the switching capability in the QS network due to changes in external 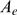 does not put severe constraints on the model. As long as an “off” state is available at low 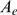 concentrations, a sufficient increase in 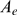 will always lead to a switch to an “on” state, due to the 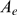 dependent increase of the constant term in Equation 1 (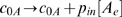, cf. Figure 1C and Equation 8 in Methods).

In nature, however, the QS switching is more restricted since it is the bacteria themselves that produce the autoinducers and there is an upper limit of how high the concentrations of 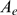 can reach within the colony. Furthermore, the 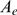 switch threshold needs to be reached while the bacteria are still in the “off” state. The production of AHL cannot be so high as to allow a single-cell to switch by itself, but it must be high enough, so that at high enough densities the colony is able to reach the threshold in 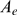.

A simplified equilibrium analysis of Equations 1–5 including a single external 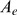 compartment, but multiple cells, leads to a single change from the non-AHL-transport analysis above. The 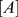 dependent term is changed: 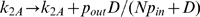, where 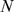 is the number of bacteria and 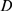 is the diffusion out from the extracellular milieu (see Methods). As discussed above, an increase of the 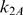 parameter moves the state of the bacteria towards a stable “off” state, upwards in Figure 1C, and this simplified model shows that adding diffusive interaction with the extracellular domain can only drive the bacteria towards that monostable “off” state.

The change due to the addition of transport is bounded (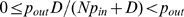), wherein the lower bound is for 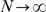. Thus the effect of increasing the population size, 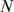, corresponds to decreasing 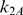 or going downwards in Figure 1C. However, since the contribution is bounded from below, the system can never move beyond, or below, the initial state. Hence, this simplified equilibrium analysis predicts that, in order to have a QS response of the colony, the parameters must be chosen such that a single bacterium without AHL transport is in an “on” state, but close enough to the multistable region to allow inclusion of the transport terms to “move” the single bacterium into its “off” state.

**Figure 2:**
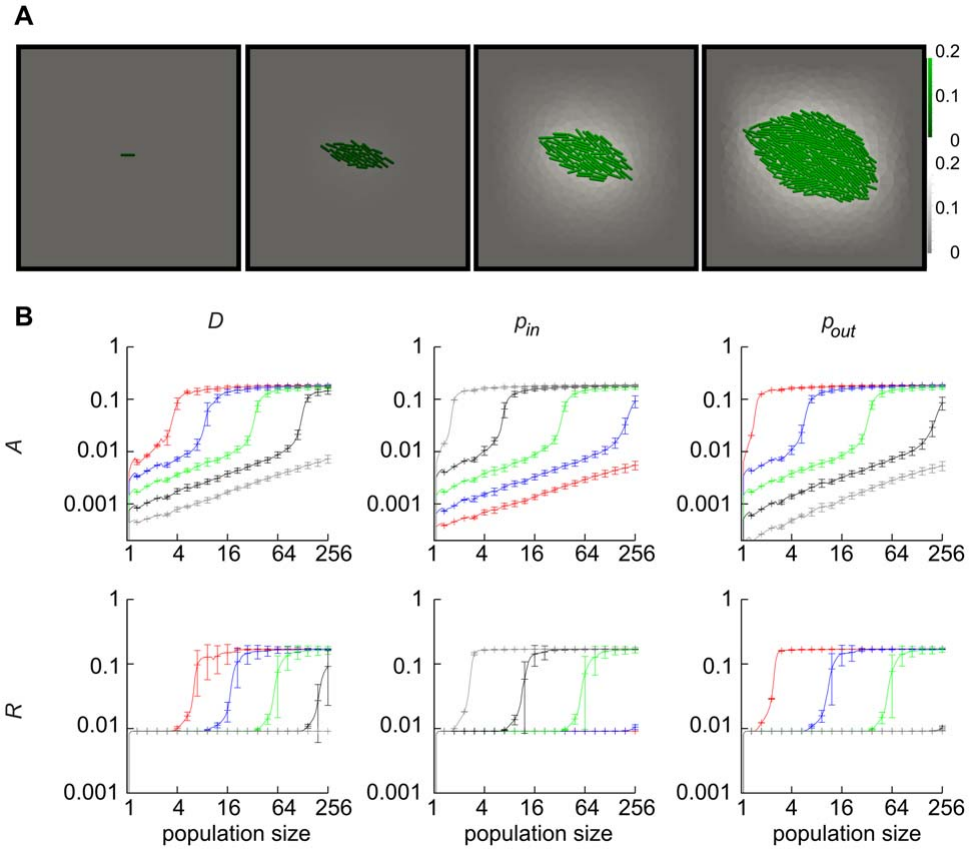
Characterization of quorum sensing responses in a simulated growing cell colony. **A**) Simulation of a dynamically growing colony. The gray colorscale refers to concentration of 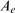 in the background and the green colorscale refers to the concentration of 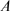 in the bacteria. There is a clear switch in 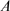 once a certain population size is reached. **B**) Parameter scans around the parameter set **P1** (Suppl. Table S1). Top panel shows 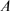 and bottom panel shows 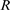 as a function of population size in the simulations when the three transport parameters, 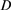 (left panel), 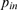 (middle panel), and 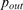 (right panel) are varied. All plots show mean values of the population with standard deviations. The green lines correspond to the original parameter set. Parameters are modified by multiplication by 0.1 (red), by 0.3 (blue), by 3 (black lines), and by 10 (gray). For the other parameters see Suppl. Figures S3, S4, S5.

The analysis presented is of course for a very simplified description of the QS, and to be able to investigate how QS works in a more realistic non-equilibrium environment, we used a cell-based model and a spatially meshed extracellular domain with dynamically diffusing AHL. We simulated the system of growing communicating bacteria, starting with a single bacterium that grows, divides and communicates with the environment (see Methods). The overall simulation domain was assumed to be a thin rectangular layer, of the same thickness as the bacteria, and with 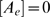 on the boundary. This assumption, in addition to simplifying visualization and analysis of the results, corresponds to the experimental design used to analyze bacterial colony growth in microfluidic devices, thus providing a potential model validation platform [27]. The colony displayed a quorum-sensing behavior with a clear unanimous switch in 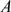 and 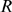 at a specific population size (Figure 2A, see also Figure S2 and Video S2). When the number of cells was small the colony was in an “off” state. At a threshold population size, cells in the spatial center of the colony started to switch on, leading to a short time period of an inhomogeneous colony with cells both in “on” and “off” states, but the switching propagated quickly and soon virtually all cells of the colony were switched on. A reason for the homogeneity of responses of all the cells in the colony is the positive feedbacks ensuring that the production of AHL is much higher in cells that are turned on compared to the constitutive basal production. As soon as a few cells turn the signaling on, the amount of AHL in the environment can quickly rise, driving the fast propagation of the switching throughout the colony. However, the low constant production of AHL is necessary for the initiation of the switching behavior.

To investigate the system dependence on the model parameters, we performed a parameter scan and studied how parameter variation affected the colony behavior (Figures S3, S4, S5). In Figure 2B we show the effect of varying the three transport parameters (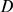, 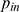, and 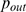). We observed that the colony response moves into and out of the bistability region at different population sizes. Specifically, we found that, at low diffusion rates, the system was inclined to switch, whereas at higher diffusion rates the colony was no longer able to accumulate sufficient amount of AHL to make the switch possible, gray line in Figure 2B. This is in accordance with the simplified equilibrium analysis above, wherein changing 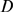 affected 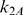 via 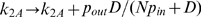. Hence, at low 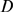, the system is essentially in the situation without AHL transport, whereas at high 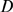, we get 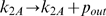 which might be enough of a change in the effective AHL removal rate to move the system into the “off” state. From the same simplified model it is also clear that 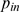 and 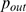 should effectively change 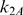 in opposite directions, which is also exactly what one observes in Figure 2B.

### The model predicts local clustering and external geometry dependent behavior

Thus far we have shown that the switching mechanism of QS in individual cells is dependent on the extracellular AHL concentration, and that for a bacterial colony this concentration depends on the net loss of local 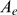. Our simplified analysis showed that this loss can be approximated by a change in 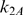, given by addition of the term 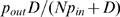, which explicitly shows that this depends on the outflux (or loss) in the exterior (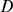) and the density of bacteria (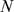), with the individual bacteria thus not being able to distinguish whether 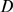 or 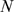 is changed in the environment. The simulations of the colony growth (Figure 2) also showed that being in the center of a dense population facilitates the QS switch. Taken together, these results demonstrate that the model confirms that bacteria cannot measure cell density, exterior loss of autoinducer, and spatial clustering independently, in agreement with prior qualitative arguments [15].

**Figure 3:**
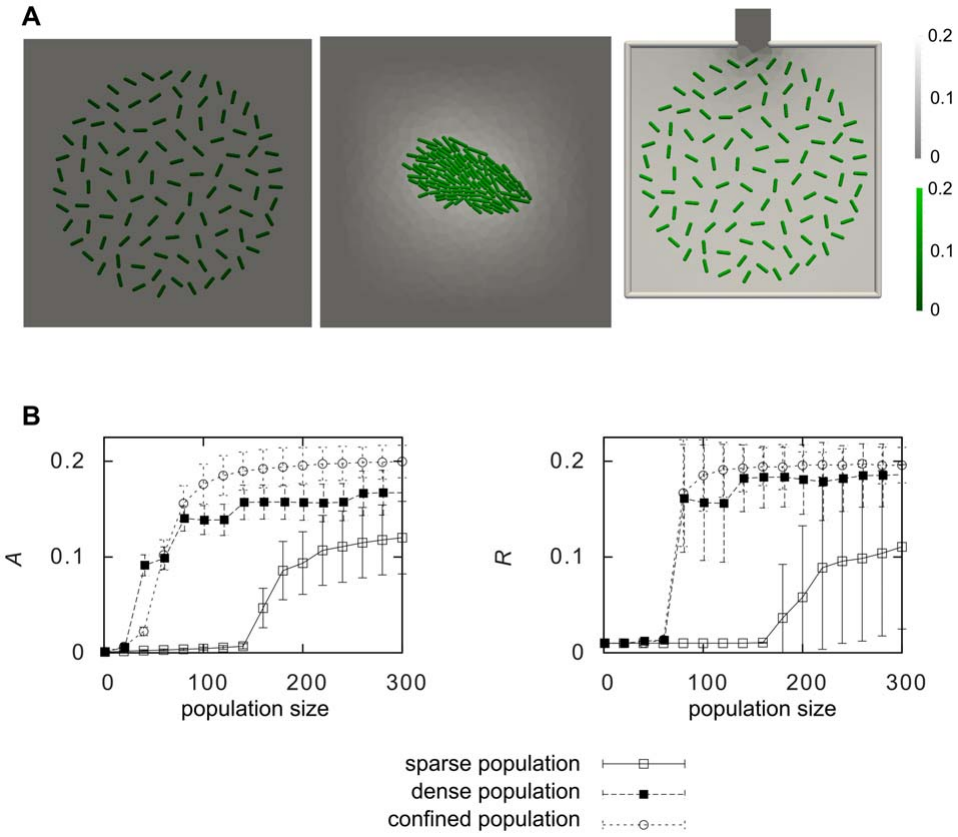
Static simulations with different degrees of local clustering and external confinement geometry. **A**) Examples of simulations of non-growing colonies of population size 100. In the left image the bacteria are positioned to give a sparse population, in the middle image they are positioned to form a very dense colony, whereas in the right image the sparse population is geometrically confined with just a small outlet. In the two latter cases the colony has switched the response on, whereas in the left image the colony remains in the “off” state. **B**) 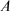 (left) and 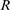 (right) as a function of population size for simulations of non-growing colonies. Mean values of the population with standard deviations are plotted. The system switches once the colony reaches a certain number of cells. For dense and confined populations the switch occurs earlier than in sparse populations.

It has been shown that bacteria often actively seek out small cavities and populate them to very high densities [28], [29]. To see how local density and confinement might affect colony behavior, we compared dense cell population simulations with simulations of a sparsely populated colony. The populations were simulated with open boundaries as before (Figure 3A). We also considered colonies confined in a small cavity with a single small outlet (Figure 3A). In the simulations, we fixed the population size at different values and examined the resulting QS. The system switched once the population reached a certain number of cells, and the system switch occurred at lower cell number in the dense population than in the sparse population (Figure 3B, see also Figure S6). This result demonstrated that although QS is generally a population-size effect, it can be facilitated by local clustering of bacteria [15]. We also observed that confinement of the sparse population makes its switching behavior similar to that of the dense population. Thus, not only the local density and the number of bacteria matters for the response of the colony but also the geometry of the surrounding environment.

**Figure 4:**
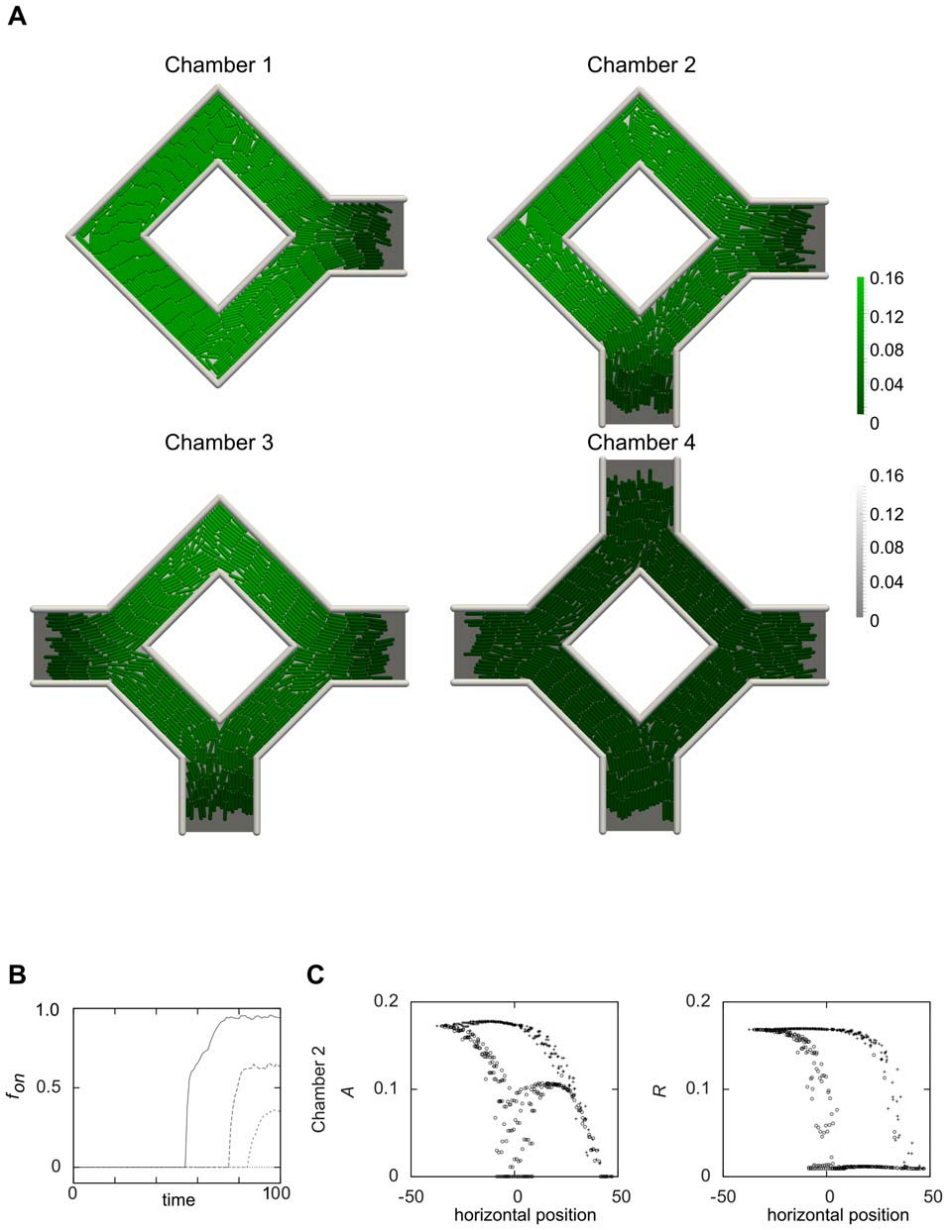
Simulations of growing cell colonies in spatial confining environments. **A**) Gray colorscale refers to autoinducer concentration in the background, and green colorscale refers to the autoinducer concentration within the cells. The figure illustrates the effect the external geometry has on the QS response of the colony. The chambers are referred to in the text as Chamber 1 (top left), Chamber 2 (top right), Chamber 3 (bottom left), and Chamber 4 (bottom right). (See also Suppl. Videos S3, S4, S5, S6.) **B**) The fraction of cells in the on state as function of time for the four different chambers. The curves are from top to bottom, Chamber 1, 2, 3, and 4. **C**) 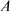 and 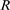 as a function of the horizontal position of the bacteria in Chamber 2. Crosses (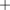) are from the upper half of the chamber and circles () are from the lower half. Data are from the last time point where the chamber is filled and the system has reached quasi-equilibrium.

In light of the results in Figure 3B the strategy of populating cavities makes sense as a way of facilitating the onset of quorum sensing. However, the geometry of the cavity may also affect the ability of the colony to perform the switch in concert, e.g. by controlling the escape of AHL. To address this possibility more directly, we performed simulations of colony growth and QS in a cavity geometry similar to previously used microfluidic chambers [27], but with variable number of outlets. Simulations were initiated with a single bacterium and simulations were run until the expanding colony completely filled up the cavity (Figure 4A and Videos S3, S4, S5, S6). At sufficiently high values of 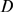 the population only partially switched states (Figure 4A). Typically, it is only at the regions furthest away from the exits that the colony was able to accumulate sufficient levels of 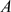 to undergo the switch. Figure 4B shows the fraction of cells in the “on” state as a function of time, with the clear result of an organized population-dependent behavior. At first no cells are in the on state, but at a colony size determined by the number of outlets, parts of the population make a sudden sharp switch and reaches a new stable configuration (cf. Videos S3, S4, S5, S6). The bacteria furthest away from the exits are those that initiate the switching behavior.

In Figure 4C the 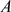 and 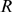 concentrations of individual bacteria are plotted as a function of the spatial position along the horizontal axis for Chamber 2. At positions far away from the exits the bacteria are homogeneously in the “on” state, while closer to the exits the population is less homogeneous due to the loss of AHL at the exits (the upper leg marked with 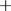 in Figure 4C). Note also that the signaling in the chamber legs between two exits is tightly concentrated around 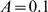 (e.g.  in Figure 4C). The system can show multi-stable responses and the cells in these legs are clearly at the stable fixed point wherein the 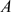 production has switched on while the 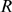 production has not. Taken together our simulations show a complex behavior with the switching of each bacterium being dependent on its location within the chamber, and with local subpopulations with high signaling homogeneity created.

### In mixed populations cheater cells have a local transient advantage

In natural habitats, bacteria live in environments with a mixture of different bacterial strains. This property can affect the QS behavior and lead to a problem of emergence of cheater cells that can exploit the “common good” produced by the QS population. The phenomenon was recently studied in controlled environments for bacteria [21], [22]. These cheater cells do not produce the autoinducer (or other QS resulting common good molecules) themselves but do take advantage of the metabolically expensive QS signaling by the rest of the population. By not participating in the generation of QS response, cheater cells can instead use metabolic energy to more rapidly grow and divide. We considered this situation by modeling cheater cells as the other *bona fide* signaling cells, but with no production of 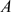 (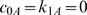). Furthermore, we assumed that once the normal cells switch on and thus increase their QS response, their growth rate slows down (see Methods).

**Figure 5:**
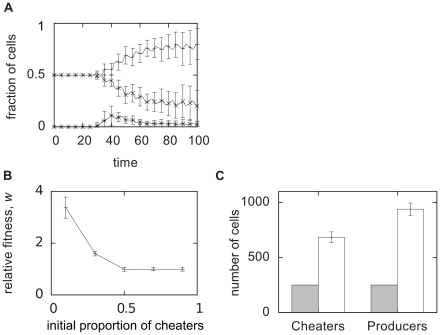
Analysis of the mixed cheater-producer populations. **A**) Statistics from 20 simulations started with different initial conditions with three producing and three cheater cells in Chamber 1 of Figure 4. Line: fraction of cheater cells, dashed: fraction of autoinducer producing cells, dash-dotted: fraction of the producing cells that is turned on. An example simulation is shown in Suppl. Video S7. Simulations from Chambers 2–4, behave similarly, see Suppl. Figure S7. **B**) Relative fitness of the cheater population as a function of the initial proportion of cheater cells. Mean values with standard deviation from 10 randomly initiated simulations are shown. At small initial proportions the cheater cells display a considerable growth advantage, whereas for larger initial proportions this advantage vanishes. **C**) Total population change in the same simulations. Gray boxes show the initial and white boxes show the final population sizes. Even though the cheater cells have a relative fitness larger than 1 in each simulation, the total fraction of producer cells increases.

Data from simulations in a confining chamber starting from different initial states are presented in Figure 5A where we tracked the population dynamics in the mixed colonies. Initially the growth rates of the producer and cheater sub-populations were equivalent, but once some of the producer cells switched states, the cheater cell population rapidly started to dominate the chamber (cf. Video S7). Note that although the fraction of producing cells that turned on was quite small (about 10%, dashed-dotted line in Figure 5A), this was sufficient to break the symmetry and give the cheater cells a clear advantage. The domination of cheater cells leads to a dilution of producing cells which lowers the AHL concentration in the chamber. This resulted in a decrease in the number of producer cells that were switched on and thus diminished the advantage of the cheater cells. In the end the relative cell numbers of the two sub-populations can stabilize. The simulations in the other chambers displayed similar behaviors (see Figure S7).

The dynamics of the colonies (Figure 5A) clearly showed that whether or not the cheater cells were at an advantage, depended on the composition of the mixture of cheater cells and normal cells [21]. To investigate this further we performed simulations wherein the initial colony consisted of different ratios of cheater and producer cells. In these simulation we added the assumption that the producing cells could provide the population with some advantage or “common good”, a property beneficial for the survival and growth of the population as a whole. In the model this was simplified by assuming an autoinducer dependent growth rate (see Methods). In Figure 5B the resulting relative fitness of the cheater cells is displayed, indicating decreasing advantage for increasing initial ratios of cheater cells, as seen in experiments [21].

The model predicts that the advantage of cheater cells is directly related to the number of producing cells that are in the “on” state, which in turn is dependent on the number and location of the cheater cells. This leads to an effective negative feedback, so that the producer cells are not completely overtaken by the cheater cells in any of the cases in Figure 5B. In fact, the addition of the AHL-dependent growth does not alter the relative fitness behavior in the individual simulations (data not shown) but actually leads to a total increase of producer cells if all initial colony configurations are summed up (Figure 5C). Although cheater cells always have a local advantage and never grow slower than the producing cells, the colonies with more producer cells will grow faster and this is sufficient for generating more producer cells in total. This has recently been reported for synthetic bacteria strains and is referred to as the Simpson's paradox [22]. The simulations with mixed populations show that cheater cells may have a local advantage, but a negative feedback via the colony growth and dilution of producing cells leads to a situation where this advantage is only transient.

## Discussion

Quorum sensing is a key example of the ability of unicellular bacteria to act not only as individual cells but also as an ensemble, resembling in many respects a multicellular organism. This collective cell behavior phenomenon is important for various biological behaviors, with considerable implications for the physiology and pathology of plants and animals [13]. Hence it merits further understanding both for a better appreciation of the fundamental properties of cell-cell communication and for its applications.

With the increasing amount of quantitative data for the molecular networks at the center of the cellular QS signaling, the use of mathematical models has emerged as an important tool for understanding how the molecular network structure with its multiple feedbacks can explain the complex behavior of the population. Previous models have mainly discussed the intracellular network with the underlying QS switch, and have treated the extracellular environment as a boundary condition [23]–[26]. An exception is the static model briefly described in Hense et al. 2007 [15]. Recent development of microscopy techniques together with the increased use of microfluidic devices have increased the ability to study cell colony behaviors at a cellular resolution [30]. Here we have presented a model explicitly taking into account individual growing bacteria as well as the transport and geometry of the extracellular milieu. This resulted in a model framework with the results directly comparable with data from cell-based experiments in microfluidic devices and other experimental settings, and allowed for an explicit investigation of how population-level behavior emerges from single-cell mechanisms. In this report, we presented simulations investigating cell-to-cell variations in homogeneous populations as well as the behavior of mixed populations.

An equilibrium analysis of the model was used to find the parameter values capable of population-size dependent QS switching and the analysis highlighted the differences between a situation where autoinducer levels are tuned extracellularly and when bacteria themselves are the only source of the autoinducer. In the former case, we showed that QS switching was not very constrained. However, in the latter case, the effect of adding the autoinducer transport boiled down to variation of a single parameter of the model: the effective degradation of the autoinducer. The variation of the effective degradation was shown to be dependent on the transport parameters characterizing the autoinducer and the cell medium, and on the number of bacteria present, and was shown to be bounded by the rate of autoinducer transport out of the cells. Hence, the ability of QS switching is only ensured if this bounded parameter can change so that the systems can visit both “on” and “off” states. A clear prediction from this analysis is that if autoinducer membrane transport is blocked, the cells would have be to be in an “on” state.

Simulations of growing and proliferating bacteria showed a population-size dependent switching behavior, wherein although it is the bacteria in the center of the colony that initially switch on, the whole colony quickly follows creating a very homogeneous behavior. This is mainly due to the strong positive feedback in the signaling system, ensuring that the autoinducer production greatly increases in the cells that are switched on. A scanning of the model parameters orders of magnitudes around their initial values showed that the main QS feature, the population switching, is very robust, while the actual population size where the switch happens is quite dependent on parameter values. We further found, as expected, that the switching of the population is driven by the external autoinducer concentration. This is dependent on the population size, but also on how much autoinducer is lost from the colony, which depends on the local density (clustering) and the confinement of the external geometry; parameters that to a large extent are beyond the control of single cells. We explored these parameters explicitly in our model simulations showing that growing dense populations in small confined cavities facilitates population switching, a potentially common strategy [28], [29]. This relates to the discussion of the evolutionary fitness advantage provided by a collective cell population behavior, with the quorum sensing, diffusion sensing and efficiency sensing have been suggested as different explanations [15]. Our model suggests that cells can sense different aspects of their environment through determination of the value of a single, albeit complex parameter (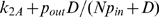), comprising all these different possibilities. Additionally, the model suggests that a possible evolutionarily selectable strategy of populating small cavities as a means to control diffusion, local density, and confinement in order to facilitate the onset of quorum sensing.

Bacteria live in environments where different biological organisms compete. It has been noted that a QS behavior can be exploited by strains of cheater cells that do not participate in some aspects of QS, but still take advantage of the benefits this provides. The corresponding advantages of this behavior for cheater cells have recently been investigated in controlled experiments [21], [22]. The cell-based approach allowed us to investigate competition between autoinducer producing and non-producing cells by adding a growth reduction for producing cells that are in their “on” state. We showed that the cheater cells did have an advantage as soon as producing cells switched on. This advantage, however, led to a dilution of producing cells, and hence the amount of autoinducer per cell, within the mixed population as the cheater cells increased their relative number. The decrease in autoinducer further led to producing cells switching “off”, which diminished the cheater cell advantage. Hence, the growth dynamics in these mixed populations creates a feedback that disallows a cheater strain to fully overtake a population. If we assumed also that the growth was dependent on the production of autoinducer or the corresponding beneficial population trait (e.g., the ability to cause the host to provide nutrients), we could observe situations where populations initiated with different ratios of cheater cells generated an overall advantage for producing cells, although in each individual local sub-population, cheater cells were never at an disadvantage. The phenomenon is known in statistics as the Simpson's paradox, and was recently demonstrated for synthetic bacterial strains [22].

The number of molecules, including members of the transcription machinery present in bacteria can be very low. Hence it is expected that effective transcription and reaction rates might be noisy, and segregation of the transcription factor molecules into the daughter cells at cell division can be inhomogeneous [31]. Interestingly, a test with complete random placement of all molecular species at division had very minor effects for the cell population (data not shown). This shows a model robustness of the population behavior to molecular fluctuations in individual cells, but it also points out a limitation of our deterministic approach. In the deterministic model, a switch from a low stable state to a high stable state does not spontaneously happen in the bistable region. Hence, to get a switch in the simulations a change of condition (e.g. increasing the number of cells) will need to move the system into the monostable high region of the state space. A fluctuation in concentrations at division will then quickly move back to the only stable state. In a stochastic model, on the other hand, it could be enough to be in the bistable region where switching between high and low states could be initiated by fluctuations in concentrations. Given the number of bacteria, external compartments, and reactions in our simulations, a complete stochastic treatment may be out of reach, but an interesting future improvement would be to add stochasticity to the model, for example via adding noise terms to the ODEs.

Recent experimental developments have changed our ability to quantify cell states, from the population averages to the dynamics of single cells. The presented work is important since it represents the same development for the mathematical models used to analyze cell-based behavior. The combination of high-resolution experiments where colonies are grown in regulated environments, and models where single cells are growing to form colonies will help understanding of how population dependent behaviors, such as quorum sensing, can be derived from single cell molecular networks.

## Methods

### Model of mechanics and growth

Following earlier efforts [27], [32] each cell is modeled as an individual object, described as two semi-spheres attached at opposite sides of a cylinder. The dynamics of the bacteria is governed by a potential 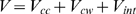, where the different contributions describe *cell-cell interactions*, *cell-wall interactions* and the *internal potential* respectively. We further assume that the dynamics of the colonies is dominated by viscous friction so the equations of motion for a given cell 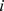 is described by
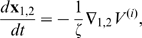
where 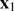 and 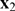 are the two coordinates, chosen as the centers of the two semi-spheres, 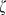 is the friction coefficient and 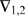 denotes the derivative with respect to 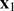 and 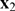 respectively. For the friction coefficient, 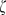 we assume a generalization of *Stokes' formula* [33]

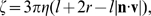
where 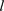 is the distance between the sphere-centers, 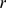 is the radius of the sphere, 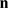 is the unit direction of the main axis of the bacterium and 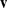 is the unit direction of its velocity.

For the cell-cell interaction and the cell-wall interaction we use an excluded volume like potential where the potential is given by
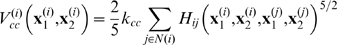
where 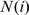 denotes the set of neighbors to cell 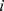 and 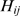 is the linear overlap between a cell 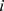 and a cell 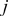 [34]. The interactions with the chamber walls are modeled in the same way, but with the only difference that the walls are assumed to be static.

The internal potential is a spring potential which is introduced to allow the coordinates to be treated as two separate degrees of freedom.
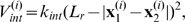
where 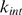 is a constant and 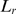 is the rest length.

The cells grow exponentially along the symmetry axis according to,
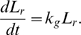
As the cells grow the intracellular molecular concentrations will decrease because of dilution. This dilution corresponds to an extra degradation term in Equations 1–4 with degradation constant 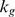.

Once a cell reaches a certain threshold length, it divides into two cells of almost the same size. At each cell division we introduce some randomness in order to break the axial symmetry of the system, giving two daughter cells with slightly different sizes and imperfect alignment [27].

### Model of cell communication

The cell-surrounding medium is modeled explicitly by dividing the space into small elements. The autoinducer molecule, 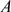, can penetrate the cell walls of the bacteria, which is modeled as a flux of 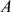 given by
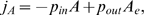
where 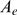 is the concentration of the element enclosing the center-of-mass of the bacterium. The contributions to the derivatives are given by
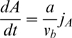


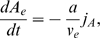
where 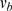 and 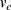 are the volumes of the bacterium and the element respectively and 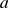 is the surface area of the bacterium.

The diffusion in the extracellular medium domain is modeled via *Fick's law* with a finite-difference version of the normal diffusion equation, the derivative of an element 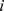 is given by
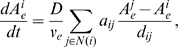
where 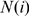 denotes the neighbors to element 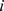, 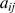 is the area between elements 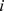 and 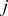 and 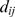 is the distance between the two elements.

### Autoinducer dependent growth

The quorum-response of the colony typically leads to the production of some “common good” or trait that is beneficial to the population as a whole, In the simulations leading up to Figure 5B–C we simplify this somewhat by having a direct autoinducer dependence in the growth function,
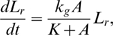
where we set 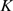 slightly below the peak value of 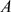, in Figure 5B–C we use 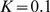. We use the same value to define if a cell is “on” or “off”, thus cells with 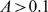 are considered to be in their “on” state. In order to model the cost of autoinducer production, we multiply the growth-rate of all the “on” cells of the system by a factor 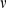, 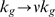. In Figure 5A we use 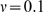 and in Figure 5B–C we use 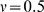.

The relative fitness measure of Figure 5B, is defined as
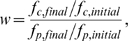
where 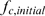 is the initial fraction of cheater cells, 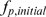 is the fraction for producer cells, and 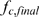 and 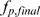 are the fractions at the end of the simulation.

### Implementation

Numerical simulations where done using an in-house developed C++ software package specifically developed to handle proliferating cells and background compartments. The differential equations are numerically solved using a fourth-order Runge-Kutta solver [35]. The software is available upon request.

### Finding stationary solutions

In order to obtain the equilibrium behavior we set Equations 1–4 to zero. This leads to two coupled equations,
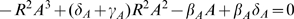


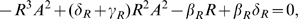
where 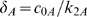, 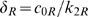, 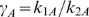, 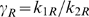, 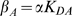, 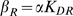 and 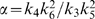, which can be combined into
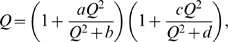
(6)where 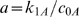, 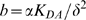, 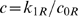, 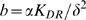, 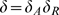. Equation 6 is obtained by setting 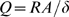, where 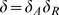, and the equation is solved numerically by finding the roots to
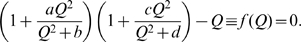
(7)We took advantage of the fact that 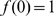 and that 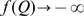 as 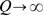 by bracketing the solutions starting by choosing two small regions, one around 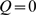 and one around a sufficiently big value of 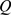. We extended these regions until 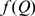 had different signs at each endpoint. This provided us with two regions where it was known that Equation 7 had solutions which could be found by a simple bisection search. By comparing the two solutions we knew whether Equation 7 had one or several solutions, see Figure 1B

Equation 6 had grouped parameters to parameterize the equilibrium solutions using only four parameters. We also used the original parameters from the model, and described the equilibrium solutions to Equations 1–4 with a similar equation
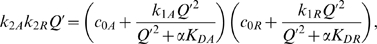
(8)where 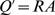 and 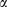 again is defined as 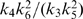. Equation 8 was solved numerically in the same way as discussed for Equation 6 above, to generate the bifurcation diagram in Figure 1C.

### Simplified equilibrium analysis

To address the effect of the autoinducer transport into and out of the extracellular environment and to examine the effect of multiple bacteria in the system we considered two simplified cases: (i) assuming a constant external 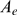 concentration, leading to a change given by 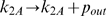 and 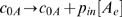 in Equation 8 and (ii) a simplified description using a single background compartment and assuming 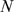 identical bacteria, with a constant transport of 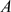 out of the compartment leading to
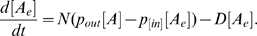
This leads to the equilibrium condition
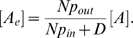
The change compared to the single-cell analysis is thus given by
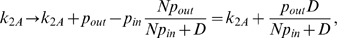
where 
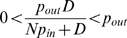
 (The lower bound comes from 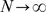 and the upper from 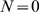). At low 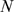 the transport will lead to a movement towards the monostable off region, i.e. for a situation with few cells the transport can lead to that a cell (which without transport would have been in its on state) is off. The effect of increasing the population size, 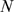, will have the opposite effect, moving it back towards the monostable on region. However, since the contribution tends to zero in the limit of big population sizes, we can never move beyond the single-cell case with no transport. This means that we must choose our single cell parameters in the monostable high region if we want a quorum-sensing response of the system.

## Supporting Information

Figure S1 Colonies display hysteretic behavior in response to changes in external autoinducer level. Shown are the mean concentrations of A (left) and R (right) as a function of the external autoinducer concentration Ae for the tree parameter sets: P1 (upper), P2 (middle), and P3 (lower) (Suppl. Table S1). Data are from simulations of a growing colony with bacteria removed once they are outside the simulation boundary, keeping the number of bacteria approximately constant (Suppl. Video S1). The volume of the extracellular medium is assumed to be much larger than that of the bacteria, resulting in negligible effect of cell-produced autoinducer. Standard deviations are smaller than the symbols used in graphs.
(0.06 MB EPS)

Figure S2 Simulation of a dynamically growing colony. The gray colorscale refers to concentration of Ae in the background and the green colorscale refers to the concentration of R in the bacteria.
(0.44 MB TIF)

Figure S3 Parameter scans around the parameter set P1 for D, k_1R_, k_1A_,k_2R_, and k_2A_. Left panel shows A and right panel shows R as a function of population size. All plots show mean values of the population with standard deviations. The green lines correspond to the default parameter set, P1. Parameters are modified by multiplication by 0.1 (red lines), by 0.3 (blue lines), by 3 (black lines), and by 10 (gray lines).
(0.17 MB EPS)

Figure S4 Parameter scans around the parameter set P1 for c_0R_, c_0A_, k_3_,k_4_, and k_5_. Left panel shows A and right panel shows R as a function of population size. All plots show mean values of the population with standard deviations. The green lines correspond to the default parameter set, P1. Parameters are modified by multiplication by 0.1 (red lines), by 0.3 (blue lines), by 3 (black lines), and by 10 (gray lines).
(0.17 MB EPS)

Figure S5 Parameter scans around the parameter set P1 for k_6_, K_DR_, K_DA_,p_in_, and p_out_. Left panel shows A and right panel shows R as a function of population size. All plots show mean values of the population with standard deviations. The green lines correspond to the default parameter set, P1. Parameters are modified by multiplication by 0.1 (red lines), by 0.3 (blue lines), by 3 (black lines), and by 10 (gray lines).
(0.17 MB EPS)

Figure S6 A (left) and R (right) as a function of population size for simulations of nongrowing colonies. Mean values of the population with standard deviations are plotted for the three different parameter sets (P1 - top, P2 - middle, and P3 - bottom) (Suppl. Table S1). The system switches once it reaches a certain number of cells. For dense and confined populations the switch happens earlier than in sparse populations.
(0.07 MB EPS)

Figure S7 Statistics from 20 simulations started with different initial conditions using equal number of producing and cheater cells in the four chambers. Line: fraction cheater cells, dashed: fraction producing cells, dash-dotted: fraction of the producing cells that is turned on.
(0.13 MB EPS)

Table S1 The three parameter sets P1, P2 and P3 used throughout the article. Results for P1 are presented in the main text, while results for the other two are presented in Suppl. Figures S1 and S6. The parameters below the lines are not part of the parameter sets but other growth and transport parameters. D*: in the simulations leading to Figure 4 we used D = 5.0.
(0.04 MB PDF)

Video S1 Simulation of a growing bacterial colony where the external Ae is slowly increased and the decreased. The bacteria are removed once they are outside a certain boundary, keeping the number ofbacteria roughly constant. Volume of the background is assumed much greater than that of the individual bacteria, thus making the contribution from the bacteria to the external Ae negligible.
(6.14 MB MOV)

Video S2 Movie from a simulation of dynamically growing colony. Gray colorscale refers to Ae in the background and green colorscale to A in the bacteria.
(0.44 MB MOV)

Video S3 Movie from simulation of colony growing in Chamber 1. Gray colorscale refers to Ae in the background and green colorscale to A in the bacteria.
(1.35 MB MOV)

Video S4 Movie from simulation of colony growing in Chamber 2. Gray colorscale refers to Ae in the background and green colorscale to A in the bacteria.
(1.16 MB MOV)

Video S5 Movie from simulation of colony growing in Chamber 3. Gray colorscale refers to Ae in the background and green colorscale to A in the bacteria.
(1.03 MB MOV)

Video S6 Movie from simulation of colony growing in Chamber 4. Gray colorscale refers to Ae in the background and green colorscale to A in the bacteria.
(0.91 MB MOV)

Video S7 Movie from simulation of colony growing in Chamber 1 starting with three producer cells (red) and three cheater cells (blue).
(1.41 MB MOV)
