# Supplementary material for: A Cell-Based Model for Quorum Sensing in Heterogeneous Bacterial Colonies
Source: PLoS Comput Biol. 2010 Jun 17;6(6):e1000819. doi: 10.1371/journal.pcbi.1000819 (PMC2887461; doi:10.1371/journal.pcbi.1000819)
Supplement: Table S1 — The three parameter sets P1, P2 and P3 used throughout the article. Results for P1 are presented in the main text, while results for the other two are presented in Suppl. Figures S1 and S6. The parameters below the lines are not part of the parameter sets but other growth and transport parameters. D*: in the simulations leading to Figure 4 we used D = 5.0. (0.04 MB PDF) [file pcbi.1000819.s008.pdf]

**Suppl. Table S1, Model parameters**

| | **P1** | **P2** | **P3** |
|---|---|---|---|
| $p_{in}$ | 0.1 | 0.1 | 0.1 |
| $p_{out}$ | 0.1 | 0.1 | 0.1 |
| $c_{0A}$ | $10^{-4}$ | $10^{-4}$ | $10^{-4}$ |
| $c_{0R}$ | $10^{-4}$ | $10^{-4}$ | $10^{-4}$ |
| $k_{1A}$ | 0.002 | 0.002 | 0.005 |
| $k_{1R}$ | 0.002 | 0.002 | 0.005 |
| $k_{2A}$ | 0.01 | 0.01 | 0.01 |
| $k_{2R}$ | 0.01 | 0.01 | 0.01 |
| $K_{DA}$ | $2 \cdot 10^{-7}$ | $10^{-6}$ | $10^{-6}$ |
| $K_{DR}$ | $10^{-4}$ | $10^{-6}$ | $10^{-6}$ |
| $k_3$ | 0.1 | 0.1 | 0.1 |
| $k_4$ | 0.1 | 0.1 | 0.1 |
| $k_5$ | 0.1 | 0.1 | 0.1 |
| $k_6$ | 0.1 | 0.1 | 0.1 |
| $D^*$ | 0.1 | 0.1 | 0.1 |
| $k_g$ | $1 \cdot 10^{-3}$ | $1 \cdot 10^{-3}$ | $1 \cdot 10^{-3}$ |
| $k_{cc}$ | 10 | 10 | 10 |
| $k_{cw}$ | 100 | 100 | 100 |
| $k_{int}$ | 10 | 10 | 10 |
| $L_{div}$ | 5.0 | 5.0 | 5.0 |
| $r$ | 0.5 | 0.5 | 0.5 |

Table 1: The three parameter sets **P1**, **P2** and **P3** used throughout the article. Results for **P1** are presented in the main text, while results for the other two are presented in Suppl. Figures S1 and S6. The parameters below the lines are not part of the parameter sets but other growth and transport parameters. $D^*$: in the simulations leading to Figure 4 we used $D = 5.0$.
